# Regulatory variation within 3’UTR of STAT5A correlates with sudden cardiac death in Chinese populations

**DOI:** 10.1080/20961790.2021.1895410

**Published:** 2021-07-23

**Authors:** Huan Yu, Yadong Guo, Zhenzhen Yang, Qing Zhang, Jiabin Xu, Qi Yang, Yiling Qu, Rui Tan, Lijuan Li, Yan He, Chengtao Li, Suhua Zhang, Bin Luo, Yuzhen Gao

**Affiliations:** a Department of Forensic Medicine, Medical College of Soochow University, Suzhou, China; b Department of Forensic Science, School of Basic Medical Sciences, Central South University, Changsha, China; c Public Security Bureau of Taixing, Taizhou, China; d Department of Epidemiology, Medical College of Soochow University, Suzhou, China; e Shanghai Key Laboratory of Forensic Medicine, Academy of Forensic Science, Shanghai, China; f Faculty of Forensic Medicine, Zhongshan School of Medicine, Sun Yat-sen University, Guangzhou, China

**Keywords:** Forensic sciences, forensic genetics, STAT5A, 3’UTR, rs3833144, sudden cardiac death, indel polymorphism

## Abstract

Definitive diagnosis to sudden cardiac death (SCD) is often challenging since the postmortem examination on SCD victims could hardly demonstrate an adequate cause of death. It is therefore important to uncover the inherited risk component to SCD. Signal transducer and activators of transcription 5 A (STAT5A) is a member of the STAT family and a transcription factor that is activated by many cell ligands and associated with various cardiovascular processes. In this study, we performed a systematic variant screening on the *STAT5A* to filter potential functional genetic variations. Based on the screening results, an insertion/deletion polymorphism (rs3833144) in 3’UTR of *STAT5A* was selected as the candidate variant. A total of 159 SCD cases and 668 SCD matched healthy controls was enrolled to perform a case-control study and evaluate the association between rs3833144 and SCD susceptibility in Chinese populations. Logistic regression analysis showed that the deletion allele of rs3833144 had significantly increased the SCD risk (odds ratio (OR) = 1.54; 95% confidence interval (CI) = 1.18–2.01; *P* = 0.000955). Further genotype-expression eQTL analysis showed that samples with deletion allele appeared to lower expression of *STAT5A*, and *in silico* prediction suggested the local 3 D structure changes of STAT5A mRNA caused by the variant. On the other hand, the bioinformatic analysis presented that promoters of *RARA* and *PTGES3L-AARSD1* could interact with rs3833144, and eQTL analysis showed the higher expression of both genes in samples with deletion allele. Dual-luciferase activity assays also suggested the significant regulatory role of rs3833144 in gene transcription. Our current data thus suggested a possible involvement of rs3833144 to SCD predisposition in Chinese populations and rs3833144 with potential function roles may become a candidate marker for SCD diagnosis and prevention.

## Introduction

Sudden cardiac death (SCD) refers to a sudden and unexpected death or arrest attributed to a cardiovascular cause, mostly occurring within 1 h from the onset of symptom or within 24 h of last been observed to be alive if not witnessed [1]. As a leading cause of mortality, SCD accounts for half of all cardiovascular deaths with an annual incidence of 57.3–110.8 per 100 000 in USA and 40.7 per 100 000 in China [2–4]. As work continues to pathological research, coronary heart diseases have been identified as the major etiology for SCD in older people [5], and inheritable arrhythmogenic diseases were chara­cterized as an important cause in young [6, 7]. Nevertheless, a definitive diagnosis to SCD is still challenging since postmortem examination could hardly demonstrate an accurate cause of death [8]. To uncover the molecular mechanisms of SCD and identify genetic markers, numerous efforts have been put into molecular genetics of SCD during the last few decades [9, 10]. For instance, recent study has identified a causative variant within caveolin-3 (Cav-3) gene in a patient with SCD [11]. Population studies also suggested a familial aggregation of SCD, characterizing the SCD family history as an impor­tant risk factor for sudden death [12–14]. The casual rare variants with large effect size to SCD may show incomplete penetrance, while common pathogenic variants with small effect size may also generate the clinical picture of SCD. Therefore, it is noteworthy to aggregate common pathogenic variants to establish polygenic risk score analyses for SCD.

Signal transducer and activators of transcription (STATs) are a family of cytoplasmic transcription factors, activated by Janus kinases (JAKs) through tyrosine phosphorylation and translocated into nuclear after dimerization [15, 16]. These activated STAT proteins regulate expression of target genes, implicated in renin-angiotensin system (RAS) [17], hypertrophy [18], angiogenesis [19, 20], fibrosis [21, 22] and other cellular processes in the cardiac myocytes. STAT5A is one member of the family and one of two highly homologous but non-redundant isoforms (STAT5A and STAT5B) [23]. Previous studies [24, 25] have identified that STAT5A of JAK/STAT pathway would be selectively activated in ischemia/reperfusion (I/R) injury and post-infarction remode­ling. Moreover, a recent study determined that STAT5A was a target of miR-222, which could hamper neovascularization and suppress atherosclerotic disease progression [20]. Considering the functional role of STAT5A in various cardiovascular processes, it merits deciphering the variants on *STAT5A* gene to explore the potential effect of STAT5A on cardiac lethal events. The 3’untranslated region (3’UTR) is the noncoding part of mRNA, harbour various functional elements and play an important role in gene regulation [26]. We therefore screened polymorphisms within this region of *STAT5A* and performed a case-control study, finally identifying an insertion/deletion (indel) variation within *STAT5A* 3’UTR correlated with SCD susceptibility in Chinese populations. Further functional experiments were implemented to investigate underlying mechanisms.

## Methods and materials

### Study populations

A total of 159 SCD cases and 668 healthy controls were enrolled in this case-control study. All the enrolled subjects were genetically unrelated Han Chinese. The 159 SCD patients were recruited from 2012 to 2020 at Sun Yat-sen University, Soochow University, Academy of Forensic Science, China and Xiangya Medical University. The recruitment criteria for both cases and controls were the same as those described previously [27, 28]. Blood samples from SCD patients were assessed by exhaustive toxicological examinations to rule out toxic death. Comprehensive forensic pathological examinations were also conducted in all the cases and different extents of coronary atherosclerosis were detected in the corpses. Except these lesions, no other mortal pathological changes were observed during the autopsy. These deceased were thus assumed to suffer sudden death derived from coronary heart diseases. The 668 healthy controls were selected through community nutritional questionnaires performed in the same regions and during the same periods as the victims. They were all frequency matched to SCD victims based on age (±5 years) and gender and identified without any cardiovascular disease history or SCD family history. This study was approved by the Ethical Committee of Soochow University. All participants or their relatives provided written informed consents.

### Genotyping of STAT5A Indel polymorphism

TIANamp Blood DNA Kit (TIANGEN, Beijing, China) was employed to extract genomic DNA from peripheral blood samples. The primers used for rs3833144 polymorphism were 5′-TGAAGCGGTCGTGTTGTGA-3′ (Forward), 5′-CACATCCCAGGACTGCACA-3′ (Reverse), which were generated by Genewiz Company (Suzhou, China). Genotyping analysis of PCR products amplified using above primers was completed with 7% non-denaturing polyacrylamide gel electrophoresis and silver staining [29]. For quality control, 50 random DNA samples were used for validation of genotyping method by means of direct sequencing. Meanwhile, 10% masked random samples were analyzed twice by independent technicians to confirm 100% reproducibility.

### Bioinformatic analysis

Mutations within *STAT5A* 3’UTR were collected from dbSNP database [30]. The genotyping and mRNA expression data available online were employed for genotype-expression analysis in our study. The RNA sequencing data of 445 lymphoblastoid cell lines was available from 1000 Genomes Project (1KGP) database (https://www.ebi.ac.uk/arrayexpress/experiments/E-GEUV-3/). The genotypes of the variants observed in 2 504 samples in 1KGP database were directly obtained from Ensembl Genome Browser (http://asia.ensembl.org/Homo_sapiens/Info/Index). Based on above data, expression quality trait loci (eQTL) analysis was performed in R(https://www.datavis.ca/R/) to evaluate genotype-phenotype association. The eQTL and splicing quality trait loci (sQTL) analysis were also performed in Genotype-Tissue expression (GTEx) database. Linkage disequilibrium (LD) analysis was performed using LD heatmap package in R. The putative influence of insertion and deletion alleles on local 3 D structure of STAT5A mRNA was predicted using SimRNAweb (https://genesilico.pl/SimRNAweb/submit) based on a 170-base-pair region containing variant rs3833144. HaploReg [31] and ENCODE [32] database were used to perform functional annotations of the variant. The chromatin interaction partners for rs3833144 were extracted from high-throughput chromosome conformation capture (Hi-C) data in 3DIV database [33].

### Construction of reporter plasmids

A total of 210-bp or 206-bp DNA fragments including the variant rs3833144 were directly generated by Genewiz Company. These fragments were directionally subcloned into KpnI and SacI sites of pGL3-promoter vector, generating two wild type vectors containing insertion allele (pGL3-proWT-*cis/trans*) and two mutant type vectors containing deletion allele (pGL3-proMT-*cis/trans*). The sequence and direction of resultant constructs were testified by direct sequencing.

### Cell culture and luciferase reporter assay

*In vitro* experiments were performed using 293 T cell lines purchased from Shanghai Cell Bank of Chinese Academy of Sciences (Shanghai, China). The cell lines were authenticated through short tandem repeat markers and maintained in Dulbecco’s modified eagle medium with 10% fetal bovine serum and 1% penicillin-streptomycin in a humidified atmosphere of 37 °C and 5% CO_2_.

Twenty-four hours after plated in the 24-well plates, 293 T cells were transfected with allele- and direction-different reporter plasmids (about 400 ng) with the corporation of jetPRIME® transfection reagent (Polyplus-transfection®, Illkirch, France). The empty pGL3-promoter vector transfected group was performed as negative control, and approximately 20 ng SV40 vector containing Renilla luciferase gene was co-transfected in each well for normali­zation of lucife­rase activity. Twenty-four hours after transfection, cells were harvested immediately by adding into 100 mL passive lysis buffer. Firefly luciferase activi­ty was assessed by the Dual Luciferase® assay system (Promega, Madison, WI, USA) in FilterMaxF5 (Molecular Devices, San Jose, CA, USA). Each group had four duplicates and every transfection experiment was repeated at least three times.

### Statistical analysis

The *X*^2^ test was performed to measure Hardy-Weinberg equilibrium for the representative of control samples. Unconditional logistic regression with adjustment for sex and age was used to eva­luate the correlations between rs3833144 and SCD risk by estimating odds ratios (ORs) and their 95% confidence intervals (CIs). The mRNA expression levels between samples with different genotypes were compared by One-way ANOVA in eQTL ana­lysis. Student’s *t* test was performed to evaluate the difference of the luciferase activities. The statistical analyses were implemented by Statistic Analysis System software (version 8.0; SAS Institute, Cary, NC, USA), a *P*-value of less than 0.05 was considered as the criterion of statistical significance. All statistical tests were two-sided in our study. The statistical power of the current sample size was calculated using the G*Power 3.1 software [34].

## Results

### Bioinformatic screening of variants within 3’UTR of STAT5A gene

**Figure 1.**
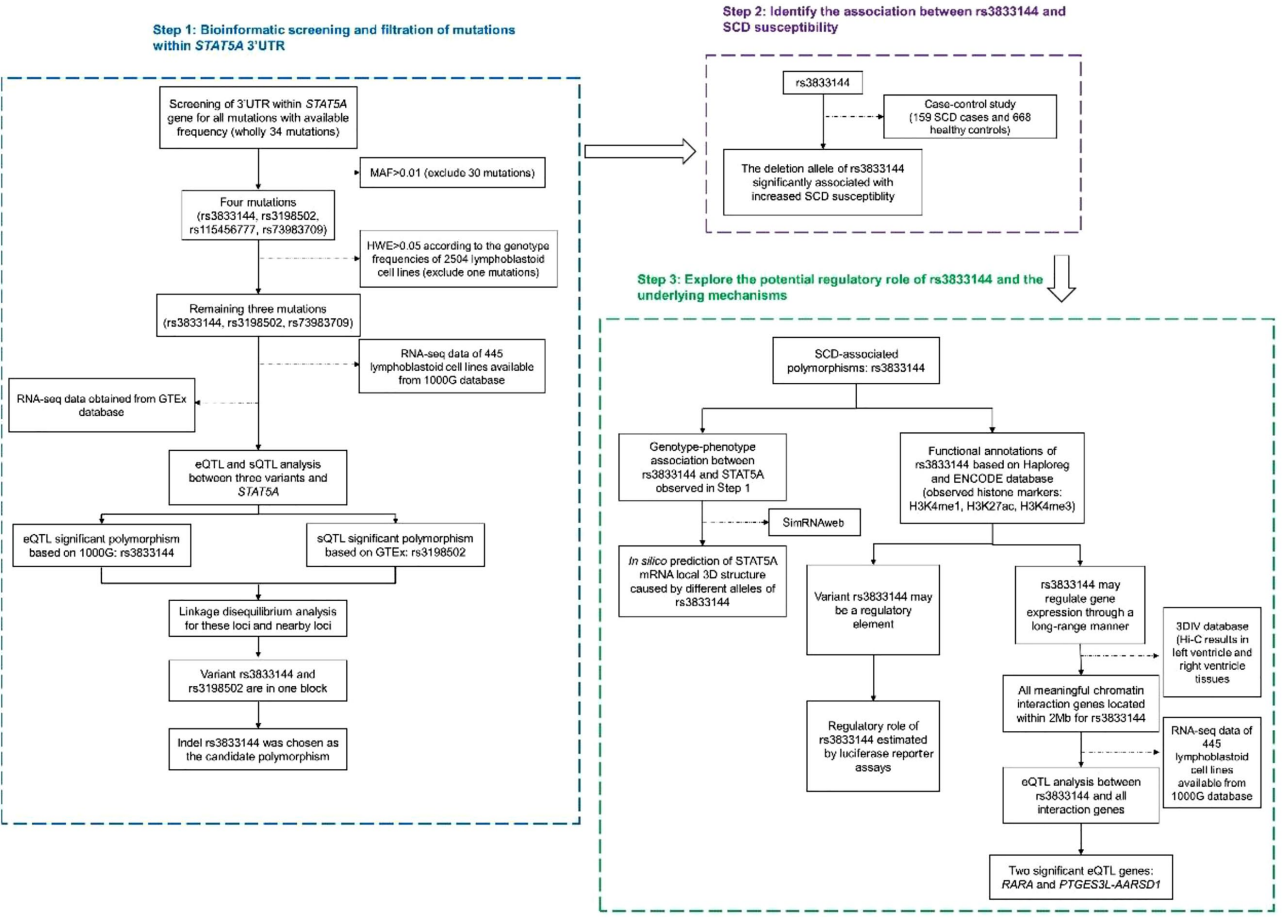
The workflow for the study on regulatory variation within 3’UTR of *STAT5A* correlates with sudden cardiac death (SCD) in Chinese populations. 3’UTR: 3’untranslated region; MAF: minimum allele frequency; HWE: Hardy-Weinberg equilibrium; GTEx: Genotype-Tissue expression; eQTL: expression quality trait loci; sQTL: splicing quality trait loci; SCD: sudden cardiac death.

**Figure 2.**
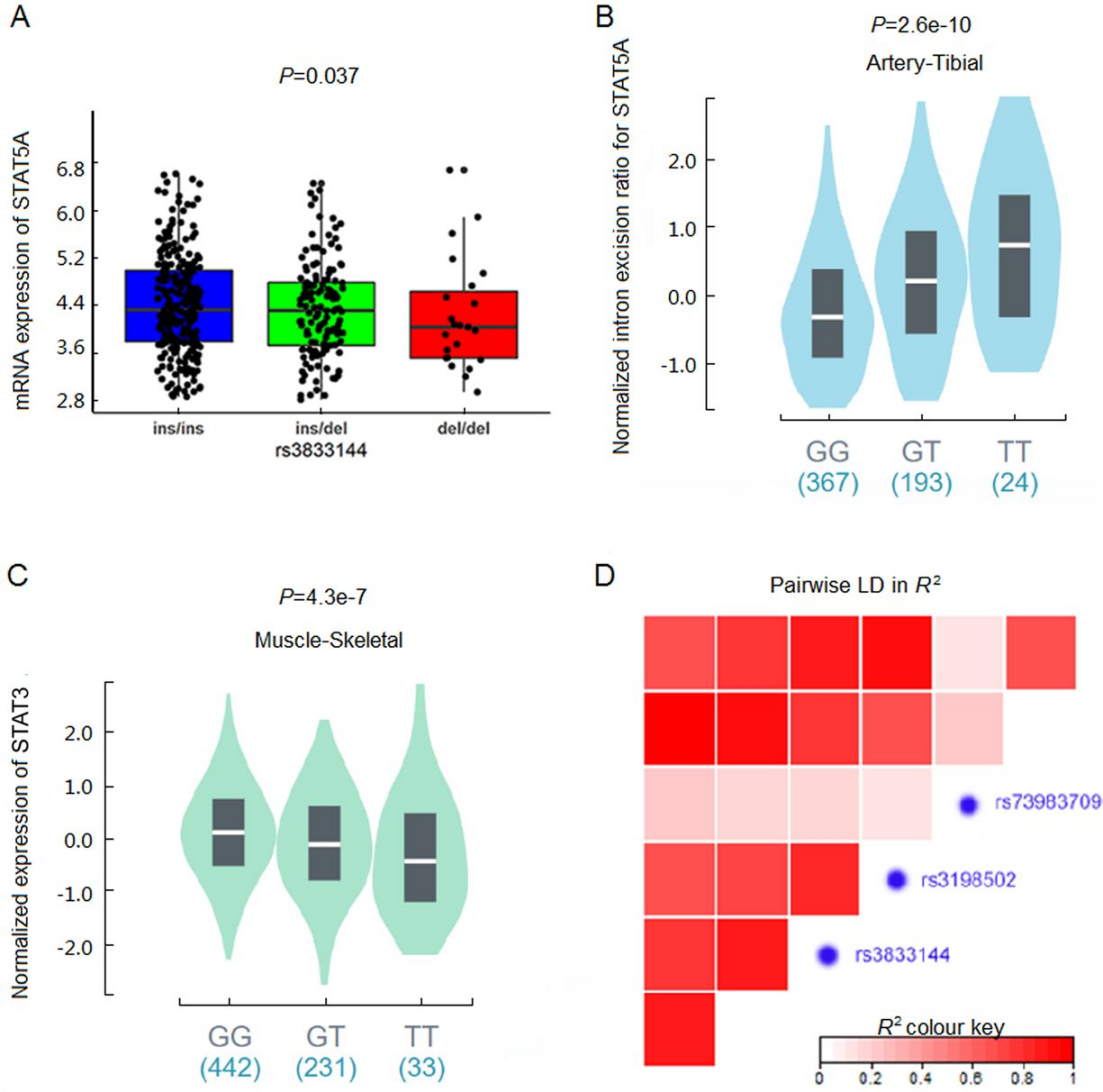
(A) Genotype-expression eQTL assay between rs3833144 and mRNA expression of STAT5A in 445 lymphoblastoid cell lines in 1000 Genomes Project database. (B) Genotype-phenotype sQTL assay between rs3198502 and STAT5A in GTex database. (C) Genotype-phenotype eQTL assay between rs3198502 and STAT3 in GTEx database. (D) Linkage disequilibrium (LD) analysis of rs3833144, rs3198502, rs73983709 and their nearby loci.

The workflow for this study was presented in Figure 1. All mutations in *STAT5A* 3’UTR with frequency available online were summarized in Supplementary Table S1. Among these mutations, only four mutations (rs3833144, rs3198502, rs115456777 and rs73983709) had a minimum allele frequency (MAF) of more than 0.01. Since rs115456777 was observed with only two genotypes, we excluded this variant to avoid potential deviation from Hardy-Weinberg equilibrium. Genotype-phenotype eQTL analysis was subsequently performed between remaining three variations and STAT5A mRNA expression levels by using genotyping and mRNA expression data from 1000 G database. As shown in Supplementary Table S2, only rs3833144 polymorphism showed a significant genotype-phenotype association with its host gene *STAT5A* (*P* = 0.037), compared with rs3198502 (*P* = 0.273) and rs73983709 (*P* = 0.096). Also, the expression levels in samples with ins/del and del/del genotypes were lower than that with ins/ins genotype, which was shown in Figure 2A. Furthermore, we observed that rs3198502 was an sQTL significant variant for *STAT5A* and an eQTL significant variant for *STAT3* based on GTEx database (Figure 2B and C), while rs72983709 was not correlated with *STAT5A* transcription. Finally, our LD analysis suggested that rs3833144 and rs3198502 were in one LD block (Figure 2D). Unlike rs3198502, indel polymorphism rs3833144 contained the characteristic of length polymorphism, which was compatible with capillary electrophoresis (CE) platform commonly applied in forensic practice. We therefore chose the indel polymorphism rs3833144 to identify its association with risk to SCD based on case-control study.

**Table 1.** Clinical characteristics of sudden cardiac death (SCD) cases and controls.

Characteristic	SCD	SCD matched controls
No. of individuals	159	668
Sex, No.		
Male	144	597
Female	15	71
Age, year, mean ± SD		
Overall	51.04 ± 13.90	49.94 ± 14.11
Males	49.87 ± 12.73	48.67 ± 12.86
Females	62.33 ± 19.36	60.61 ± 18.99
Events at sudden death (SD)		
Sleep	13	
Nonspecific	57	
Physical activity	38	
Stress	51	
Symptoms before SD		
None	92	
Others	67	
Megalothymus		
Positive	2	
Negative	157	

**Table 2.** Association between rs3833144 and sudden cardiac death (SCD) susceptibility.

Genetic model	Genotype	Cases (%)		Control (%)		OR 95%CI^a^	*P* value
Codominant model	ins/ins	64 (40.25)		344 (51.50)		1.00 (Reference)	
	ins/del	69 (43.40)		270 (40.42)		1.38 (0.93–2.04)	0.0977
	del/del	26 (16.35)		54 (8.08)		2.53 (1.41–4.51)	6.83 × 10^-4^; 7.80 × 10^-4^ (*P*trend)
							
Dominant model	ins/ins	64 (40.25)		344 (51.50)		1.00 (Reference)	
	ins/del + del/del	95 (59.75)		324 (48.50)		1.57 (1.08–2.27)	0.0124
Recessive model	ins/ins + ins/del	133 (83.65)		614 (91.92)		1.00 (Reference)	
	del/del	26 (16.35)		54 (8.08)		2.17 (1.26–3.72)	2.42 × 10^-3^
Additive model	ins allele	197 (61.95)		958 (71.71)		1.00 (Reference)	
	del allele	121 (38.05)		378 (28.29)		1.54 (1.18–2.01)	9.55 × 10^-4^

CI: confidence interval; OR: odds ratio.

a Adjusted for age and gender factors.

### Correlations between STAT5A rs3833144 polymorphism and SCD susceptibility

**Figure 3.**
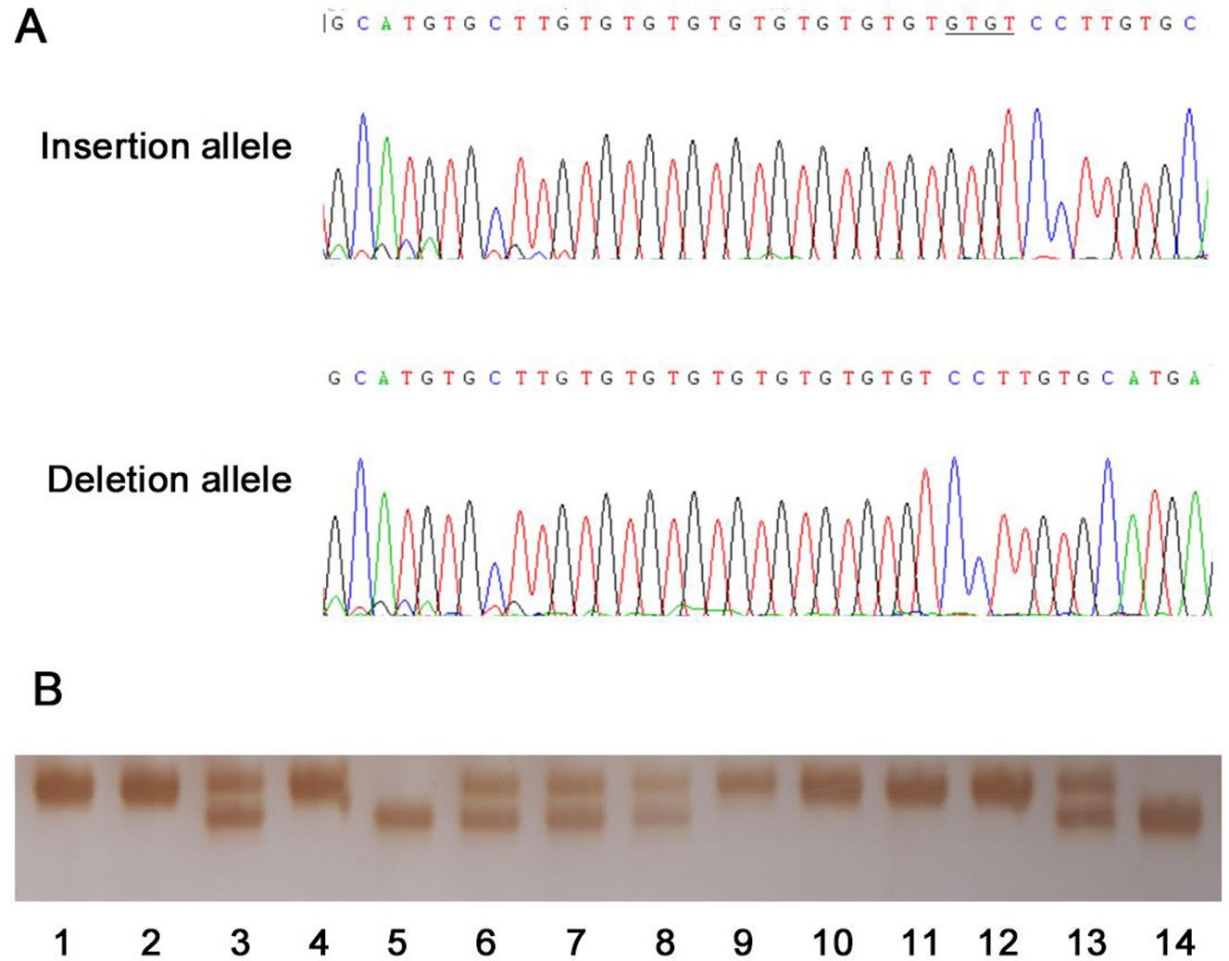
Examples of sequencing and genotyping output of rs3833144. (**A**) The sequencing output of insertion and deletion allele of rs3833144. The underlined bases are the “GTGT” insertion in coding strands. (**B**) The examples of the genotyping results. The amplified DNA fragments were analyzed by 7% non-denaturing polyacrylamide gel electrophoresis and silver staining (lane 3, 6, 7, 8 and 13, ins/del genotype; lane 5 and 14, del/del genotype; remaining lanes, ins/ins genotype

The demographic characteristics of both SCD subjects and their matching controls in present study were summarized in Table 1. The characteristics of the SCD cases are listed in Supplementary Table S3. The median age of the SCD patients was 50 years old. Among the case group, there was a remarkable discrepancy in the amount of two genders, at a male/female ratio of 9.6:1, suggesting male as a significant risk factor for SCD. More than half (58%) of the death occurred without previous symptom, and 70 cases (44%) took place without heavy physical or emotional stress, indicating that SCD was an unexpected and unpredictable disease. Examples of genotyping and sequencing output were displayed in Figure 3. The genotype frequencies of *STAT5A* rs3833144 observed in the controls were in correspondence with Hardy-Weinberg equilibrium. The G*Power 3.1 software was employed, examining a statistical power of 0.712 with *α* set at 0.05 under the dominant model.

Genotypic frequencies of rs3833144 along with OR and its 95%CI in both cases and controls were presented in Table 2. Carriers of del/del genotype significantly increased SCD risk in comparison with ins/ins genotype under a codominant model (adjusted OR: 2.53, 95%CI: 1.41–4.51, *P* = 6.83 × 10^−4^). Similar trends were also pronounced in both domi­nant and recessive model (adjusted OR: 1.57, 95%CI: 1.08–2.27, *P* = 0.0124; adjusted OR: 2.17, 95%CI: 1.26–3.72, *P* = 2.42 × 10^−3^). The 4-bp deletion allele had 1.54-fold elevated risk of SCD (adjusted OR: 1.54, 95%CI: 1.18–2.01, *P* = 9.55 × 10^−4^). As a result, these findings indicated that deletion allele of rs3833144 would contribute to a higher susceptibility to SCD.

### rs3833144 alters local 3D structure of STAT5A mRNA

Although rs3833144 locates in 3’UTR of *STAT5A* gene harbouring abundant miRNA binding sites, it was difficult to predict a matched miRNA whose binding could be interrupted by this “GTGT” indel variant, since “GT” repeats significantly abound in this locus.

**Figure 4.**
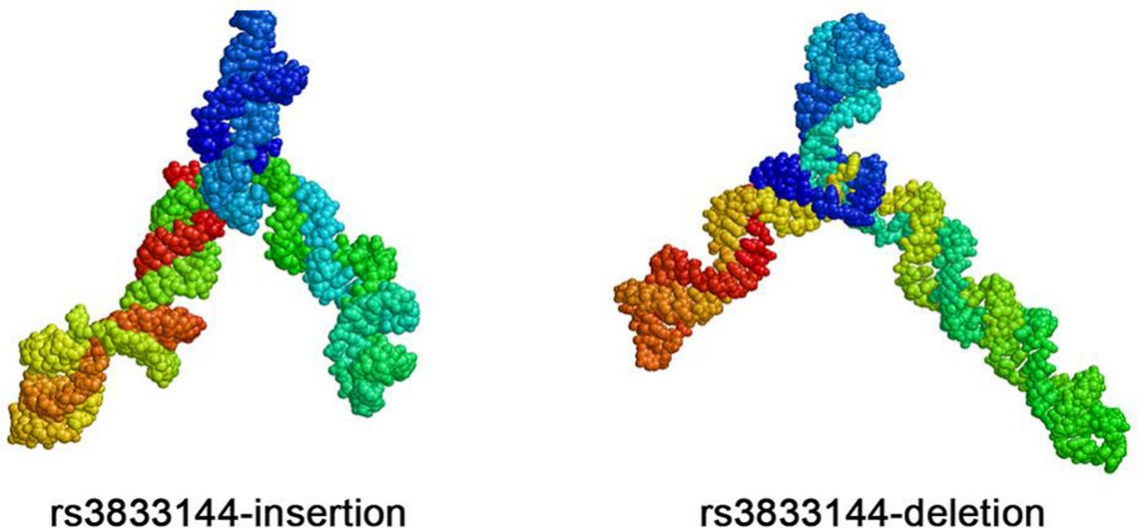
the altered local 3 D structure of STAT5A mRNA caused by insertion and deletion allele.

RNA structure plays important roles in gene expression regulation and biological function. To explore mechanisms underlying the genotype-expression association between rs3833144 and *STAT5A* presented by our eQTL analysis, we used SimRNAweb to predict local 3 D structure of STAT5A mRNA. As presented in Figure 4, the insertion of “GTGT” or not contributed to a visua­lized discrepancy in mRNA local structures. The altered local structure within 3’UTR might influence mRNA expression of *STAT5A* at a post-transcriptional level.

### Functional annotation and bioinformatic analysis of rs3833144

**Figure 5.**
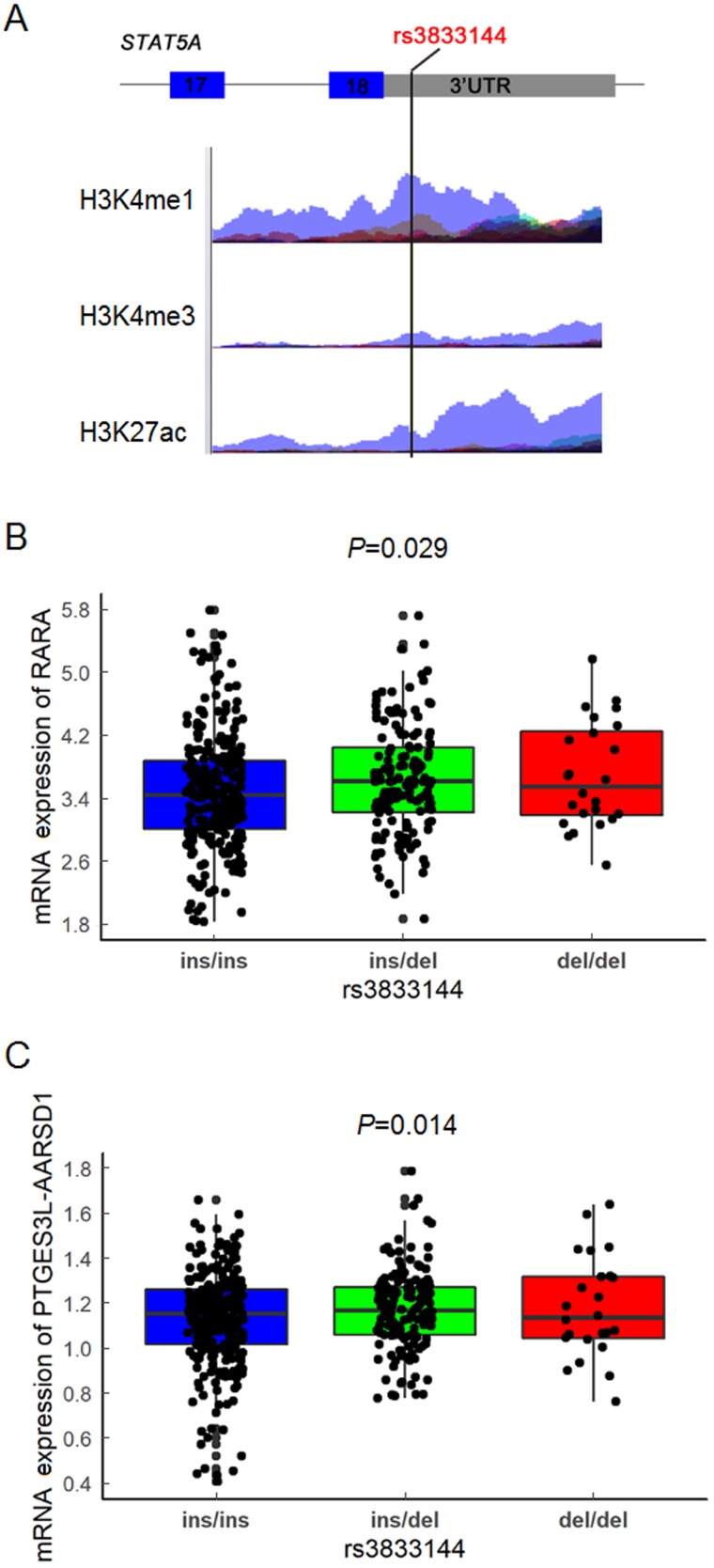
**(A)** Functional annotation of the rs3833144 loci using ENCODE database. Coloured histograms for ChIP-sequencing data containing histone modification markers (H3K4me1, H3K4me3 and H3K27ac) in seven human cell types (GM12878, H1-hESC, HSMM, HUVEC, K562, NHEK and NHLF). (B) Genotype-expression eQTL analysis between rs3833144 and mRNA expression of *RARA* and (C) *PTGES3L-AARSD1* in 445 lymphoblastoid cell lines in 1000 Genomes project database. ins/ins, *N* = 274; ins/del, *N* = 147; del/del, *N* = 24.

To further interrogate the potential regulatory roles of variants rs3833144, we next conducted *in silico* functional annotation based on Haploreg and ENCODE database. As summarized in Supplementary Table S4, among most of cell lines and tissues available in Haploreg database, the rs3833144 polymorphism located in a region with enrichment of H3K4me1 and H3K27ac and depletion of H3K4me3. Similar modification profiles can also be found in ENCODE database, which were shown in Figure 5A. These results all indicated that rs3833144 locus may reside in a long-range regulatory element. We therefore speculated that some nearby genes may be interacting with and regulated by this regulatory element, and examined all genes located within 2 Mb for interactions based on Hi-C data from 3DIV database. Two tissues, left and right ventricle, were chosen as model systems and distance normalized interaction frequency >2.00 was considered as the criterion of biologically significant interactions. The significant chromatin interaction partners (genes) for rs3833144 locus were listed in Supplementary Table S5 and S6. A total of 47 (left ventricle) and 43 (right ventricle) genes were identified to possibly interact with rs3833144 locus, and 26 genes were found in both two tissues.

**Figure 6.**
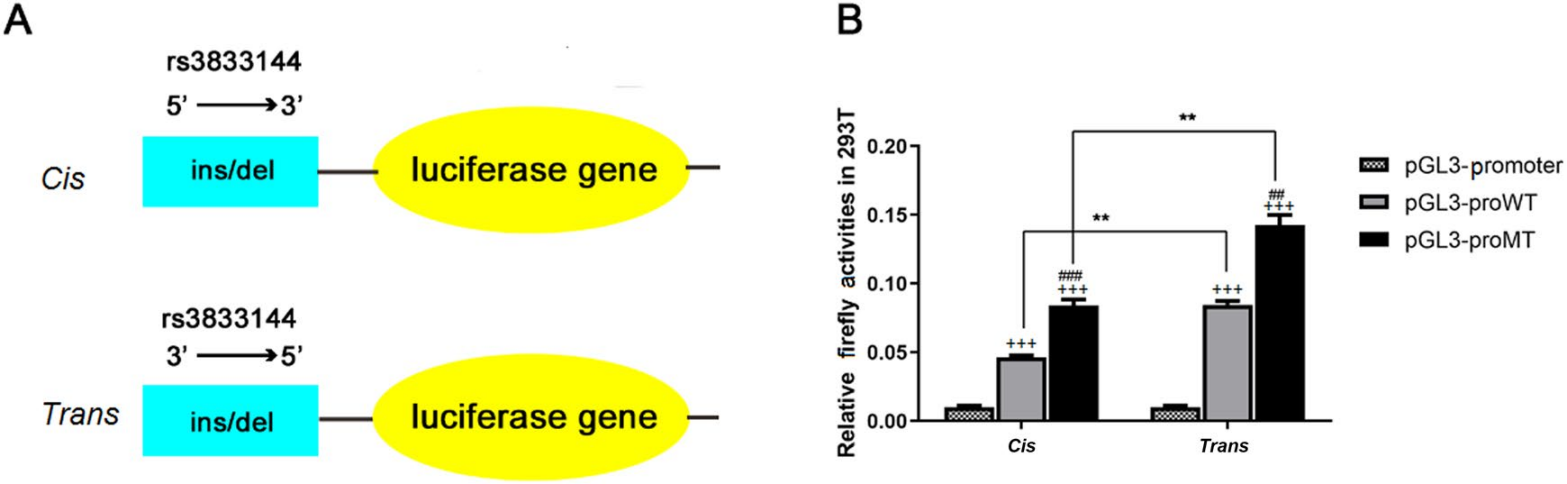
The effect of rs3833144 on gene transcriptional activity as determined by luciferase reporter assay. (**A**) plasmids used for luciferase reporter assays. DNA fragments containing variant rs3833144 (210-bp for insertion allele and 206-bp for deletion allele) were directionally subcloned into pGL3-promoter vector. (**B**) The luciferase activities were compared between construct groups containing different alleles in 293T lines, regardless of 5’ or 3’ direction of the fragments (###*P* < 0.001, compared with pGL3-proWT in *cis* group; ##*P* < 0.01, compared with pGL3-proWT in *trans* group). The luciferase activities of both insertion or deletion construct group were compared between construct groups including different directions of the fragments (**P < 0.01); Cells transfected with pGL3-proWT-*cis/trans* or pGL3-proMT-*cis/trans* showed a significantly higher luciferase activity as compared with cells transfected with pGL3-promoter (+++*P* < 0.001).

To further investigate whether the transcription of interaction genes would be influenced by rs3833144 through an allele-dependent manner, we performed genotype-expression eQTL analysis between rs3833144 and the interaction genes. Only two genes, retinoic acid receptor alpha (*RARA*) and PTGES3L-AARSD1 readthrough (*PTGES3L-AARSD1*) were identified to possess genotype-phenotype correlations with rs3833144. Intriguingly, the chromatin interactions of the two genes with rs3833144 were not found in either of the two tissues (Figure 5B). As shown in Figure 5B and C, the mRNA level of *RARA* (*P* = 0.029) and *PTGES3L-AARSD1* (*P* = 0.014) both appeared to an apparent difference between samples with different geno­types, manifesting an increasing trend in samples with ins/del and del/del genotypes compared with ins/ins genotype. These findings revealed the possibility that the rs3833144 locus might interact with the promoters of *RARA* and *PTGES3L-AARSD1* and thereby regulate the gene transcription through a long-range regulation mechanism.

### The regulatory role of rs3833144 on gene transcription activity

Based on Hi-C data and eQTL analysis, we have observed the chromatin interactions and genotype-phenotype correlations between rs3833144 and two interaction genes. To validate the effects of the indel variant on transcription activity, we next performed luciferase reporter assays using pGL3-promoter vector with allele- and direction-different fragments harbouring rs3833144 (Figure 6A). As shown in Figure 6B, the luciferase activities appeared to be significantly different between the groups transfected with vectors harbouring insertion allele (pGL3-proWT) and the groups harbouring deletion allele (pGL3-proMT), regardless of 5′ or 3′ direction of the fragments (*P*<0.01). Moreover, the constructs harbouring *trans* (3′→5′) fragments had significant higher luciferase expression than constructs harboring *cis* (5′→3′) fragments (*P*<0.01, *P*<0.001). They both had significant higher luciferase expression than fragments transfected with pGL3-prometer (*P*<0.001). These results all demonstrated the regulatory properties of rs3833144 on gene transcription.

## Discussion

In this study, a novel indel polymorphism has been identified to associate with risk of SCD through bioinformatic screening and subsequent case-control study. On the basis of our bioinformatic and functional analysis, we discovered that rs3833144 played a functional role probably through the following two manners: firstly, rs3833144 might affect 3 D structure of *STAT5A* mRNA and thereby influence its expression through a structure-dependent manner; se­condly, rs3833144 could interact with promoters of nearby genes and regulate gene transcription activities *via* a long-range mechanism. These results uncovered the possible biological mechanisms underlying the correlation between rs3833144 and SCD risk. Thus, the indel polymorphism may become a potential marker for forensic molecular diagnosis and genetic counseling of SCD.

As a signal transducer and transcriptional factor (TF), the role of STAT5A in the progression of cardiovascular diseases remains contradictory. On one hand, STAT5A would be activated by JAK2 during ischemia/reperfusion (I/R) injury and bind to the specific domain located in promoter of angiotensinogen gene, contributing to activation of cardiac renin angiotensin system (RAS) [24]. Moreover, depression of STAT5A attributed to miR-222 in endothelial cells would protect against advanced neovascularized athe­rosclerotic lesions [20]. These results suggested that STAT5A would play a pathological role in atherosclerosis and myocardial ischemia injury. On the other hand, there was also some evidence indicating the cardioprotective role of STAT5A. For instance, STAT5A was reported to be indispensable for the cardiacprotection of ischemia preconditioning (PC) against myocardial I/R injury [35, 36]. When treated with berberine, relaxin, a protein known for its anti-fibrotic effects would be endogenously upregulated because of reduced STAT3 and increased STAT5A binding to the relaxin promoter [22]. The discrepancy might be attributed to different upstream stimulations and downstream targets for STAT5A. Despite the contradictory role of STAT5A in the heart function, there is no doubt that either upregu­lation or repression of STAT5A would trigger some transient factors and cause malignant effects on cardiovascular system. In our present study, we have identified that the deletion allele of rs3833144 linked to higher SCD susceptibility, together with repression of *STAT5A* mRNA expression. Previous study have reported that genetic variants could change RNA local structure, affect the mRNA stability and regulate gene expression [37, 38], and our *in silico* ana­lysis exhibited the altered 3 D structures of *STAT5A* mRNA caused by different alleles of the variant indeed. We therefore proposed a hypo­thesis that the deletion allele of rs3833144 may impair the stability of STAT5A mRNA, which thereby contributes to aberrant suppression of *STAT5A* expression, interfere cardioprotective role of STAT5A and finally facilitate the occurrence of SCD.

SCD is a complex disease caused by various factors, such as cardiac diseases, genetic factors, drastic emotional change or exercises. Consistently, STAT5A regulation may also be interfered by various regulatory factors or genetic variants. Our eQTL analysis based on 1000 G database have identified that rs3833144 is associated with *STAT5A* mRNA expression. Apart from this variant, the sQTL analysis performed based on GTEx database also uncovered that rs3198502 may influence intron splicing of *STAT5A*, which may interfere STAT5A function. These bioinformatic results have suggested two possible regulatory factors for *STAT5A* regulation. Further, our LD analysis also shows that polymorphism rs3833144 and rs3198502 are in one LD block. Considering the contribution of rs3833144 to SCD risk, we therefore speculated that rs3833144 and rs3198502 may play a synergetic role in STAT5A regulation as well as SCD occurrence.

*Cis*-regulatory elements (CREs), such as promo­ters, enhancers, silencers, and insulators, are non-coding DNA regions which regulate gene expression at a transcriptional level. Genome-wide association studies have suggested a series of disease-associating non-coding mutants could disrupt the CREs and influence expression of nearby genes *via* a long-range mechanism [39–41]. Our functional annotations found that the region containing rs3833144 showed enrichment of H3K4me1 and H3K27ac and depletion of H3K4me3, which were all indicative markers of enhancers [42]. Further bioinformatic analysis identified that *RARA* and *PTGES3L-AARSD1* interacted with rs3833144, and eQTL analysis manifested significant genotype-expression correlations between rs3833144 and the two genes with a higher expression linked to deletion allele. The luciferase assays finally demonstrated the regulatory role of the variant on gene expression, suggesting the possibility that rs3833144 may reside in a potential CRE and control the transcription of *RARA* and *PTGES3L-AARSD1* through a long-range mechanism.

*RARA*, a member of nuclear retinoic acid (RA) receptor family, acts as a transcription factor and control gene transcription through a ligand-dependent manner. It has been reported that cardiac impairment of RARA would lead to the development of diastolic dysfunction [43], while activated RARA would protect artery from atherosclerosis by derepressing miR-10a in vascular endothelial cells in oscillatory shear regions [44]. Despite these cardioprotective role, overexpression of RARA could also stimulate differentiation of aortic endothelial cells and enhance RA-induced angiogenesis [45]. Considering that increased neovascularization within atherosclerotic plaques would enhance the risk of plaque rupture [46], *RARA* overexpression may play a pathological role in plaque rupture and finally increase SCD risk, especially to the victims with high extents of athe­rosclerosis. *PTGES3L-AARSD1* represents naturally readthrough transcription and encodes a fusion protein of two nearby genes: prostaglandin E synthase 3 like (*PTGES3L*) and alanyl-tRNA synthetase domain containing 1 (*AARSD1*). Although few studies have been reported about PTGES3L-AARSD1, this kind of transcription-induced chimera may harbour the properties of both genes [47]. *PTGES3L* encodes a protein possessing similar region with prostaglandin E synthase 3 (PTGES3), a synthase of prostaglandin E2 (PGE2). It is previously reported that PGE2 could contribute to cardiac hypertrophy [48], atherogenesis [49] and inflammatory cardiomyopathies [50]. Meanwhile, both PTGES3 (also known as p23) and AARSD1 functions as co-chaperones of heat shock protein 90 (HSP90), which participates in cardiac hypertrophy and heart failure [51]. Based on the evidence exemplified above, we could certainly specu­late that the PTGES3L-AARSD1 play a promi­nent role in cardiovascular diseases and dysregulation of PTGES3L-AARSD1 would be harmful to human heart. Taken together, it is plausible that the overexpression of *RARA* and *PTGES3L-AARSD1* derived from deletion allele of rs3833144 might cause malignant changes to our heart and participate in the pathophysiology of SCD.

Some limitations should be emphasized in our study. Since the RNA-seq and genotyping data of cardiomyocytes are not available at present, our eQTL analysis mainly focused on lymphoblastoid cell lines in 1KGP database. Therefore, based on limited bioinformatic results we can only propose possible hypotheses about how the variant rs3833144 contribute to SCD risk, further functional assays are needed to verify our results. Additionally, the significance of the finding that the deletion allele of rs3833144 contributed to the SCD risk are still limited by small sample size in our study. Further replicated case-control studies with different or expanded populations are needed to guarantee our observations. Finally, since our SCD cases mostly suffer sudden death originated from coronary atherosclerosis, our results raised the possibility that rs3833144 may be associated with coronary atherosclerosis susceptibility in natural populations. However, this implication still needs to be further investigated by coronary atherosclerosis related studies, which may have potential interests for cardiovascular community.

## Conclusion

Here, we have provided the initial evidence that the indel polymorphism (rs3833144) within the 3’UTR of *STAT5A* gene was significantly associated with SCD risk. Our current data thus suggested a possible involvement of rs3833144 to SCD predisposition in Chinese populations and rs3833144 with potential function roles may become a candidate marker for SCD diagnosis and prevention.

## References

[1] Zipes DP, Wellens HJ. Sudden cardiac death. Circulation. 1998;98:2334–2351. 9826323 10.1161/01.cir.98.21.2334

[2] Sara JD, Eleid MF, Gulati R, et al. Sudden cardiac death from the perspective of coronary artery disease. Mayo Clin Proc. 2014;89:1685–1698. 25440727 10.1016/j.mayocp.2014.08.022

[3] Benjamin EJ, Virani SS, Callaway CW, et al. Heart disease and stroke statistics—2018 update: a report from the American Heart Association. Circulation. 2018;137:e67–e492. 29386200 10.1161/CIR.0000000000000558

[4] Feng XF, Hai JJ, Ma Y, et al. Sudden cardiac death in Mainland China: a systematic analysis. Circ Arrhythm Electrophysiol. 2018;11:e006684. 30571181 10.1161/CIRCEP.118.006684

[5] Myerburg RJ, Junttila MJ. Sudden cardiac death caused by coronary Heart Disease. Circulation. 2012;125:1043–1052. 22371442 10.1161/CIRCULATIONAHA.111.023846

[6] Goff ZD, Calkins H. Sudden death related cardiomyo­pathies: hypertrophic cardiomyopathy. Prog Cardiovasc Dis. 2019;62:212–216. 31004609 10.1016/j.pcad.2019.04.001

[7] Emery MS, Kovacs RJ. Sudden cardiac death in athletes. JACC Heart Fail. 2018;6:30–40. 29284578 10.1016/j.jchf.2017.07.014

[8] Basso C, Carturan E, Pilichou K, et al. Sudden cardiac death with normal heart: molecular autopsy. Cardiovasc Pathol. 2010;19:321–325. 20381381 10.1016/j.carpath.2010.02.003

[9] Bezzina CR, Lahrouchi N, Priori SG. Genetics of sudden cardiac death. Circ Res. 2015;116:1919–1936. 26044248 10.1161/CIRCRESAHA.116.304030

[10] Magi S, Lariccia V, Maiolino M, et al. Sudden cardiac death: focus on the genetics of channelopathies and cardiomyopathies. J Biomed Sci. 2017;24:56. 28810874 10.1186/s12929-017-0364-6 PMC5556354

[11] Lariccia V, Nasti AA, Alessandrini F, et al. Identification and functional analysis of a new putative caveolin-3 variant found in a patient with sudden unexplained death. J Biomed Sci. 2014;21:58. 24917393 10.1186/1423-0127-21-58 PMC4109384

[12] Friedlander Y, Siscovick DS, Arbogast P, et al. Sudden death and myocardial infarction in first degree relatives as predictors of primary cardiac arrest. Atherosclerosis. 2002;162:211–216. 11947916 10.1016/s0021-9150(01)00701-8

[13] Jouven X, Desnos M, Guerot C, et al. Predicting sudden death in the population: the Paris Prospective Study I. Circulation. 1999;99:1978–1983. 10209001 10.1161/01.cir.99.15.1978

[14] Kaikkonen KS, Kortelainen ML, Linna E, et al. Family history and the risk of sudden cardiac death as a manifestation of an acute coronary event. Circulation. 2006;114:1462–1467. 17000909 10.1161/CIRCULATIONAHA.106.624593

[15] Ihle JN. The Stat family in cytokine signaling. Curr Opin Cell Biol. 2001;13:211–217. 11248555 10.1016/s0955-0674(00)00199-x

[16] Horvath CM. STAT proteins and transcriptional responses to extracellular signals. Trends Biochem Sci. 2000;25:496–502. 11050435 10.1016/s0968-0004(00)01624-8

[17] Satou R, Gonzalez-Villalobos RA. JAK-STAT and the renin-angiotensin system: the role of the JAK-STAT pathway in blood pressure and intrarenal renin-angiotensin system regulation. JAKSTAT. 2012;1:250–256. 24058780 10.4161/jkst.22729 PMC3670281

[18] Haghikia A, Stapel B, Hoch M, et al. STAT3 and cardiac remodeling. Heart Fail Rev. 2011;16:35–47. 20407820 10.1007/s10741-010-9170-x

[19] Osugi T, Oshima Y, Fujio Y, et al. Cardiac-specific activation of signal transducer and activator of transcription 3 promotes vascular formation in the heart. J Biol Chem. 2002;277:6676–6681. 11744720 10.1074/jbc.M108246200

[20] Dentelli P, Rosso A, Orso F, et al. microRNA-222 controls neovascularization by regulating signal transducer and activator of transcription 5A expression. Arterioscler Thromb Vasc Biol. 2010;30:1562–1568. 20489169 10.1161/ATVBAHA.110.206201

[21] Chen Y, Surinkaew S, Naud P, et al. JAK-STAT signalling and the atrial fibrillation promoting fibrotic substrate. Cardiovasc Res. 2017;113:310–320. 28158495 10.1093/cvr/cvx004 PMC5852635

[22] Gu HP, Lin S, Xu M, et al. Up-regulating relaxin expression by G-quadruplex interactive ligand to achieve antifibrotic action. Endocrinology. 2012;153:3692–3700. 22673230 10.1210/en.2012-1114

[23] Buitenhuis M, Coffer PJ, Koenderman L. Signal transducer and activator of transcription 5 (STAT5). Int J Biochem Cell Biol. 2004;36:2120–2124. 15313458 10.1016/j.biocel.2003.11.008

[24] Mascareno E, El-Shafei M, Maulik N, et al. JAK/STAT signaling is associated with cardiac dysfunction during ischemia and reperfusion. Circulation. 2001;104:325–329. 11457752 10.1161/01.cir.104.3.325

[25] El-Adawi H, Deng L, Tramontano A, et al. The functional role of the JAK-STAT pathway in post-infarction remodeling. Cardiovasc Res. 2003;57:129–138. 12504822 10.1016/s0008-6363(02)00614-4

[26] Mayr C. Regulation by 3’-untranslated regions. Annu Rev Genet. 2017;51:171–194. 28853924 10.1146/annurev-genet-120116-024704

[27] Wang S, Zhang Z, Yang Y, et al. An insertion/deletion polymorphism within 3’UTR of RYR2 modulates sudden unexplained death risk in Chinese populations. Forens Sci Int. 2017;270:165–172. 10.1016/j.forsciint.2016.12.005 27987400

[28] Yin Z, Zhang Q, Zhou W, et al. Influence of functional polymorphism in MIF promoter on sudden cardiac death in Chinese populations. Forens Sci Res. 2017;2:152–157. 10.1080/20961790.2017.1327744 PMC6197097 30483634

[29] Allen RC, Graves G, Budowle B. Polymerase chain reaction amplification products separated on rehydratable polyacrylamide gels and stained with silver. Biotechniques. 1989;7:736–744. 2483661

[30] Sherry ST, Ward MH, Kholodov M, et al. dbSNP: the NCBI database of genetic variation. Nucleic Acids Res. 2001;29:308–311. 11125122 10.1093/nar/29.1.308 PMC29783

[31] Ward LD, Kellis M. HaploReg: a resource for exploring chromatin states, conservation, and regulatory motif alterations within sets of genetically linked variants. Nucleic Acids Res. 2012:D930–D934. 22064851 10.1093/nar/gkr917 PMC3245002

[32] Davis CA, Hitz BC, Sloan CA, et al. The Encyclopedia of DNA elements (ENCODE): data portal update. Nucleic Acids Res. 2018;46:D794–D801. 29126249 10.1093/nar/gkx1081 PMC5753278

[33] Yang D, Jang I, Choi J, et al. 3DIV: a 3D-genome interaction viewer and database. Nucleic Acids Res. 2018;46:D52–D57. 29106613 10.1093/nar/gkx1017 PMC5753379

[34] Faul F, Erdfelder E, Buchner A, et al. Statistical power analyses using G*Power 3.1: tests for correlation and regression analyses. Behav Res Methods. 2009;41:1149–1160. 19897823 10.3758/BRM.41.4.1149

[35] Yamaura G, Turoczi T, Yamamoto F, et al. STAT signaling in ischemic heart: a role of STAT5A in ischemic preconditioning. Am J Physiol Heart Circ Physiol. 2003;285:H476–82. 12860560 10.1152/ajpheart.00079.2003

[36] Chen H, Jing XY, Shen YJ, et al. Stat5-dependent cardioprotection in late remote ischaemia preconditioning. Cardiovasc Res. 2018;114:679–689. 29365089 10.1093/cvr/cvy014

[37] Duan J, Wainwright MS, Comeron JM, et al. Synonymous mutations in the human dopamine receptor D2 (DRD2) affect mRNA stability and synthesis of the receptor. Hum Mol Genet. 2003;12:205–216. 12554675 10.1093/hmg/ddg055

[38] Mooers BH, Logue JS, Berglund JA. The structural basis of myotonic dystrophy from the crystal structure of CUG repeats. Proc Natl Acad Sci USA. 2005;102:16626–16631. 16269545 10.1073/pnas.0505873102 PMC1283809

[39] Pomerantz MM, Ahmadiyeh N, Jia L, et al. The 8q24 cancer risk variant rs6983267 shows long-range interaction with MYC in colorectal cancer. Nat Genet. 2009;41:882–884. 19561607 10.1038/ng.403 PMC2763485

[40] Harismendy O, Notani D, Song X, et al. 9p21 DNA variants associated with coronary artery disease impair interferon-gamma signalling response. Nature. 2011;470:264–268. 21307941 10.1038/nature09753 PMC3079517

[41] Smemo S, Campos LC, Moskowitz IP, et al. Regulatory variation in a TBX5 enhancer leads to isolated congenital heart disease. Hum Mol Genet. 2012;21:3255–3263. 22543974 10.1093/hmg/dds165 PMC3384386

[42] Mora A, Sandve GK, Gabrielsen OS, et al. In the loop: promoter-enhancer interactions and bioinformatics. Brief Bioinform. 2016;17:980–995. 26586731 10.1093/bib/bbv097 PMC5142009

[43] Zhu S, Guleria RS, Thomas CM, et al. Loss of ­myocardial retinoic acid receptor α induces diasto­lic dysfunction by promoting intracellular oxidative stress and calcium mishandling in adult mice. J Mol Cell Cardiol. 2016;99:100–112. 27539860 10.1016/j.yjmcc.2016.08.009 PMC5107335

[44] Lee DY, Yang TL, Huang YH, et al. Induction of microRNA-10a using retinoic acid receptor-α and retinoid x receptor-α agonists inhibits atherosclero­tic lesion formation. Atherosclerosis. 2018;271:36–44. 29459264 10.1016/j.atherosclerosis.2018.02.010

[45] Gaetano C, Catalano A, Illi B, et al. Retinoids induce fibroblast growth factor-2 production in endothelial cells *via* retinoic acid receptor α activation and stimu­late angiogenesis *in vitro* and *in vivo*. Circ Res. 2001;88:E38–47. 11230116 10.1161/01.res.88.4.e38

[46] Roth L, Rombouts M, Schrijvers DM, et al. Chronic intermittent mental stress promotes atherosclerotic plaque vulnerability, myocardial infarction and sudden death in mice. Atherosclerosis. 2015;242:288–294. 26233915 10.1016/j.atherosclerosis.2015.07.025

[47] Akiva P, Toporik A, Edelheit S, et al. Transcription-mediated gene fusion in the human genome. Genome Res. 2006;16:30–36. 16344562 10.1101/gr.4137606 PMC1356126

[48] Mendez M, LaPointe MC. PGE2-induced hypertrophy of cardiac myocytes involves EP4 receptor-dependent activation of p42/44 MAPK and EGFR transactivation. Am J Physiol Heart Circ Physiol. 2005;288:H2111–H2117. 15626689 10.1152/ajpheart.00838.2004

[49] Gross S, Tilly P, Hentsch D, et al. Vascular wall-produced prostaglandin E2 exacerbates arterial thrombosis and atherothrombosis through platelet EP3 receptors. J Exp Med. 2007;204:311–320. 17242161 10.1084/jem.20061617 PMC2118736

[50] Toth AD, Schell R, Levay M, et al. Inflammation leads through PGE/EP3 signaling to HDAC5/MEF2-dependent transcription in cardiac myocytes. EMBO Mol Med. 2018;10:e8536. 29907596 10.15252/emmm.201708536 PMC6034133

[51] Ranek MJ, Stachowski MJ, Kirk JA, et al. The role of heat shock proteins and co-chaperones in heart failure. Phil Trans R Soc B. 2018;373:20160530. 29203715 10.1098/rstb.2016.0530 PMC5717530

